# The responses of Ht22 cells to oxidative stress induced by buthionine sulfoximine (BSO)

**DOI:** 10.1186/1471-2202-6-10

**Published:** 2005-02-12

**Authors:** Jun Chen, Andrea Small-Howard, Amy Yin, Marla J Berry

**Affiliations:** 1 Department of Cell & Molecular Biology, John A Burns School of Medicine, University of Hawaii, 1960 East West Rd, Honolulu, HI 96822 USA

## Abstract

**Background:**

glutathione (GSH) is the most abundant thiol antioxidant in mammalian cells. It directly reacts with reactive oxygen species (ROS), functions as a cofactor of antioxidant enzymes, and maintains thiol redox potential in cells. GSH depletion has been implicated in the pathogenesis of neurological diseases, particularly to Parkinson's disease (PD). The purpose of this study was to investigate the change of cellular antioxidant status and basic cell functions in the relatively early stages of GSH depletion.

**Results:**

in this study, GSH was depleted by inhibition of glutamylcysteine synthetase using buthionine sulfoximine (BSO) treatment in Ht22, a neuronal cell line derived from mouse hippocampus. Treatment with BSO produced dose-dependent decreases in total GSH level, Fe3+-reducing ability (FRAP assay), Cu2+-reducing ability (Antioxidant Potential, AOP assay), and ABTS free radical scavenging ability (ABTS assay) of the cells, but the sensitivity of these indicators to dosage varied considerably. Most of the changes were completed during the first 8 hours of treatment. Cell viability was tested by MTT (3-[4,5-dimethylthiazol-2-yl]-2,5-diphenyl tetrazolium bromid) assay, and cells at lower density in culture were found to be more sensitive to GSH depletion. The activity of antioxidant enzymes, such as glutathione peroxidase (GPx), glutathione reductase (GR), and copper/zinc superoxide dismutase (Cu/Zn-SOD) were affected by GSH depletion. A cDNA expression array assay of the effects of BSO treatment showed significantly decreased mRNA level for 3 genes, and significantly increased mRNA level for 10 genes, including the antioxidant enzymes Cu/Zn-SOD and thioredoxin peroxidase 2 (TPxII).

**Conclusions:**

the study suggests that there are BSO-sensitive and BSO-resistant pools of GSH in Ht22 cells, and that different categories of antioxidant react differently to GSH depletion. Further, the effect of GSH status on cell viability is cell density dependent. Finally, the alterations in expression or activity of several antioxidant enzymes provide insight into the various cellular responses to GSH depletion.

## Background

Glutathione (GSH, tripeptide γ-L-glutamyl-L-cysteinyl-glycine) is the most abundant thiol antioxidant in mammalian cells. It reacts directly with reactive oxygen species (ROS), or functions as a cofactor of antioxidant enzymes such as the glutathione peroxidases (GPxs). In addition, GSH keeps sulfhydryl groups of cytosolic proteins in reduced form by maintaining thiol redox potential in cells [1], and regulates cell signaling pathway in apoptosis [2,3]. The requirement for GSH and total antioxidant capacity is particularly high in brain.

Brain consumes 20% of total oxygen in the body, and thus undergoes high levels of oxidative challenge. Cumulative oxidative damage has been strongly implicated in neurodegeneration and neurological diseases, such as Parkinson's disease (PD), Alzheimer's disease (AD), and amyotrophic lateral sclerosis (ALS). The importance of GSH in particular has been indicated in the case of PD. Among the progressive oxidative stresses that occur in the pathogenesis of PD, an characteristic is the decrease in total GSH concentrations in the substantia nigra in preclinical stages, when other biochemical changes are still undetectable [4].

Investigation of the consequences of intracellular GSH depletion in neuronal cell lines has relied predominantly on one of three methods. These are: (a) treatment with homocysteic acid (HCA) or glutamate to block the uptake of cystine, a substrate for GSH synthesis [5-7]; (b) treatment with BSO [5] to inactivate γ-glutamylcysteine synthetase, the rate limiting enzyme in GSH synthesis, and (c) treatment with ethacrynic acid [8] or diethyl maleate [9] to react with the thiol group of GSH. The oxidative stress caused by GSH depletion further affects the status of other antioxidants. The concept of total antioxidant capacity (TAC) has been developed to assess the general antioxidant activity in biological samples without distinguishing the contribution from each individual component. Measurements of TAC have been intensively developed in the past 20 years. The principle of these methods is to evaluate the total effect of all contributing compounds in the system by one criterion, such as free radical scavenging, electron donation or protection against the oxidative damage to lipids, proteins or DNA. But considering the fact that different categories of antioxidant work through different mechanisms and need specified conditions for maximal function, it is impossible to cover the activities of all antioxidants in one assay. Thus multi-dimensional measurements on TAC have been suggested [10].

In this study, GSH was depleted by BSO in Ht22, a neuronal cell line derived from mouse hippocampus. GSH level, TAC, antioxidant enzyme activity, cell viability and gene expression were assessed.

## Results and discussion

### Intracellular total GSH level *vs*. cell density in culture

**Figure 1:**
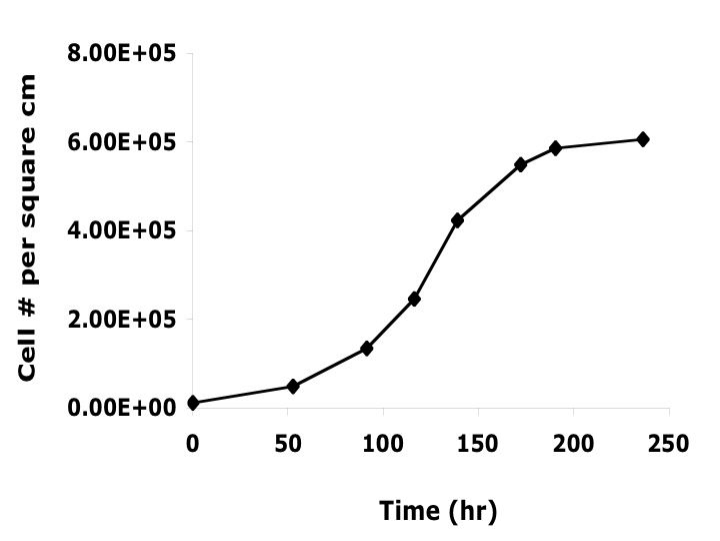
Growth curve of Ht22 cells. Cell number counted by hemocytometer, three counts for each point.

**Figure 2:**
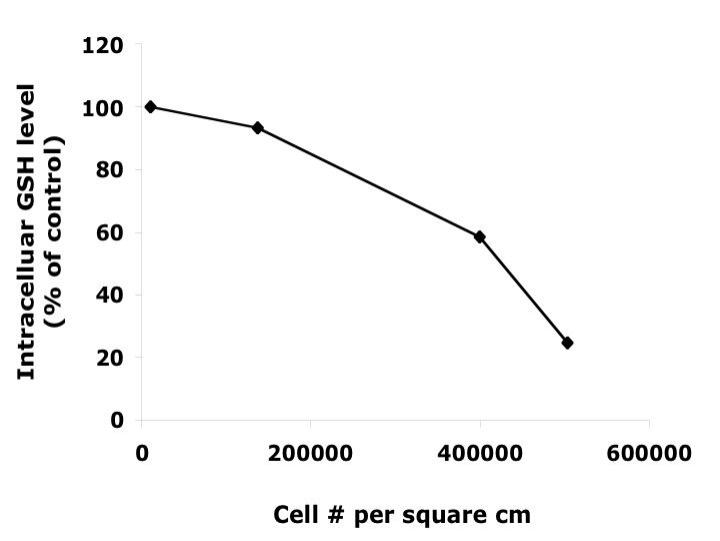
Total intracellular GSH concentration in Ht22 cells. The GSH levels decrease with increasing cell density in culture.

Before depleting GSH from cells, total GSH levels in Ht22 were monitored from pre-log phase to the end of log phase of cell growth (Figures 1 &2). Intracellular GSH levels decreased as cell density increased, with this effect being more dramatic as the cells entered log phase growth.

Whether the decrease of GSH with cell growth is due to limited nutrient supply or to programmed regulation is not known. It has been reported that the GSH content of brain cells depends strongly on the availability of precursors for GSH [11]. It was also noticed that as Ht22 cells grow denser in culture, the intracellular ROS level decreased (Chen et al., unpublished data), as apparently balances with the decreased GSH concentration.

### Dose responses

#### Intracellular GSH

**Figure 3:**
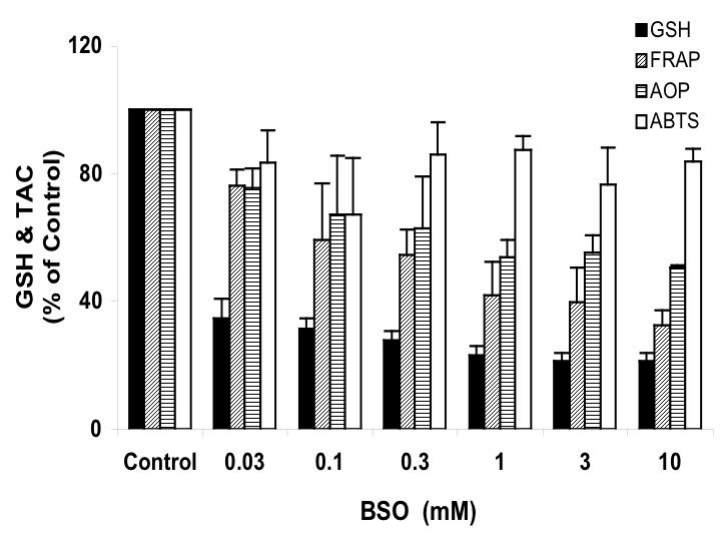
Dose response of Ht22 cells to BSO treatment. The effects of 15-hour BSO treatment on GSH level and total antioxidant capacity in Ht22 cells were measured. Average value and SD are shown, N = 3. In comparison to 0.03 mM of BSO, increasing the concentration to 1 mM or higher caused significant decreases in GSH levels (P values ≤ 0.0399).

The dose responses of Ht22 to BSO were analyzed by varying BSO concentration from 0.03 to 10 mM in a 15-hr treatment, and the changes of intracellular GSH level and TAC were measured (Figure 3). Treatment with 0.03 mM BSO resulted in a dramatic decrease of total GSH level to 35% of the control level. Increasing the BSO concentration to 10 mM further decreased the GSH level to 22% of control, representing an additional drop of only 13%. In comparison to 0.03 mM of BSO, increasing the concentration to 1 mM or higher caused significant decreases in GSH levels (P values ≤ 0.0399). This result suggests there are two pools of GSH in the cell, one easily depleted by BSO, and the other more resistant to depletion. A previous study by Seyfried et al. [12] showed that BSO treatment of PC12 cells was more efficient at depleting cytosolic GSH than mitochondrial GSH, indicating that the BSO-sensitive and BSO-resistant GSH pools in Ht22 might localize to cytosol and mitochondria, respectively.

After 10 mM BSO treatment for 15 hrs, the predominant form of glutathione was the reduced form (GSH); only about 5% of the total glutathione was found in the oxidized form (GSSG). After 15 hrs BSO treatment, malondialdehyde (MDA) assay showed the increases of Abs at 586 nm caused by MDA formation were: control = 0.011 ± 0.006; 1 mM BSO = 0.012 ± 0.006; 3 mM BSO = 0.009 ± 0.004; and 10 mM BSO = 0.011 ± 0.004 (average ± SD, 3–5 independent experiments), no increase in lipid peroxidation after the treatments was observed, indicating GSH depletion at these levels was not yet destructive to the cells.

#### Total antioxidant capacity

Three methods were employed in this study to investigate antioxidant status following BSO treatment of Ht22 cells. The Cu2+-reducing ability assay (Antioxidant Potential, AOP) and Fe3+-reducing ability assay (FRAP) both measure the activity of metal ion-reducing antioxidants, but the FRAP assay is characterized by its low pH (3.7), thus excluding the antioxidant function of thiols. Figure 3 shows that from 0.03 to 10 mM BSO treatment, the FRAP value had a sharp decrease from 76 to 32% of the control, indicating that some non-thiol antioxidants were expended to preserve the BSO-resistant GSH or to cope with oxidative stress caused by the depletion of BSO-sensitive GSH. In comparison to FRAP, the AOP value showed a gradual decrease from 76 to 50 % of control through the range of BSO concentrations, and this lesser decrease may be partially maintained by the BSO-resistant GSH pool in the cells.

In contrast to FRAP and AOP, the ABTS free radical scavenging ability (ABTS assay) of the cells maintained at about 80% of the control level at all BSO concentrations. Cao et al. [13] previously showed that GSH is highly reactive to ABTS radical. Unlike the FRAP and AOP assays, the ABTS assay appears to be less sensitive to the metal ion-reducing antioxidants that were consumed by GSH depletion.

#### Cell viabilities and bioreduction activity

##### Cell viabilities

**Figure 4:**
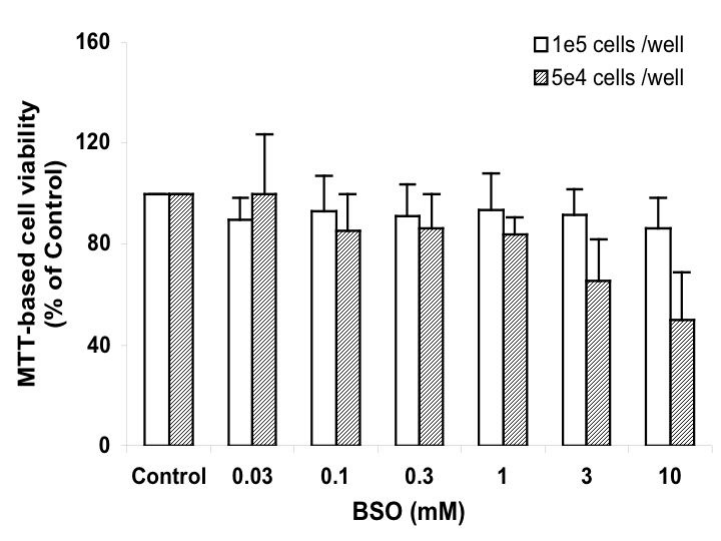
MTT assay for the viability of Ht22 cells treated with BSO for 15 hours. Average value and SD are shown (1e5 cells/ well, N = 9; 5e4 cells/ well, N = 5). At 3 and 10 mM BSO concentrations, the differences caused by cell density are significant (P values ≤ 0.0027).

Effects of GSH depletion on cell viability were assayed using the MTT (3-[4,5-dimethylthiazol-2-yl]-2,5-diphenyl tetrazolium bromid)-based cell viability assay, an indicator of mitochondrial activity (Figure 4). Ht22 cells at higher density (1e5 cells per well, equal to 1.3e5 cells per square cm) were more resistant to GSH depletion, and in spite of the change in BSO concentration, the decrease in cell viability remained at about 10% of control. Lower cell density (5e4 cells per well, equal to 6.5e4 cells per square cm) rendered higher sensitivity; as BSO concentration increased from 0.03 to 10 mM, the viability dropped from 100 to 50% of control. At 3 and 10 mM BSO concentrations, the differences caused by cell density are significant (P values ≤ 0.0027). These results show that the mitochondrial activity of cells is influenced by GSH depletion, and that this effect is sensitive to the density of cells in culture. The reason(s) behind the cell density factor is not clear. Either proliferation status or communication between cells may contribute. In neurodegenerative diseases, the effect of cell death, thus the decrease of cell density, on the susceptibility of neurons to GSH depletion will be worth investigating. Wither sever cell death can accelerate GSH loss is not known.

No changes in cell viability in response to BSO treatment were detected by trypan blue staining, which distinguishes dead cells from live ones. Cell viabilities were 98.9% (N = 25) for untreated cells, and 99.6% (N = 5), 99.1% (N = 5), and 98.1% (N = 12), after 15 hrs treatment with 1, 5, and 10 mM BSO, respectively. The oxidative stress produced in these treatments is not sufficient to be lethal to the cells, in accordance with the MDA assay, which shows no increase in lipid peroxidation.

##### Bioreduction activity

**Figure 5:**
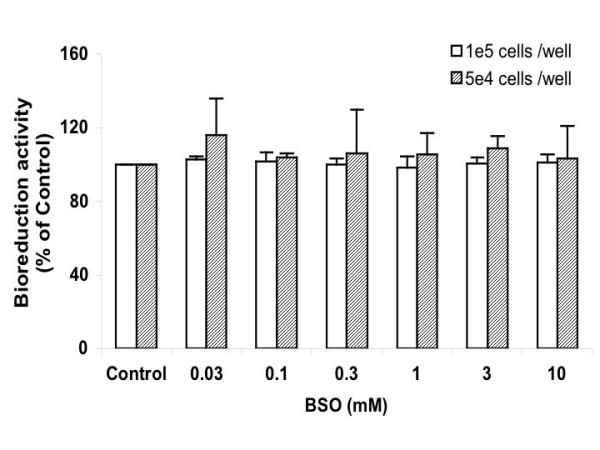
Resazurin based bioreduction assay of Ht22 cells treated with BSO for 15 hours. Average value and SD are shown (1e5 cells/ well, N = 6; 5e4 cells/ well, N = 5). For 1e5 cells/well, treatment of 0.03 mM BSO caused significant increase in the bioreduction activity *vs *control (P = 0.0053); for 5e4 cells/well, the increases caused by treatments of 0.1 and 3 mM BSO were significant vs control (P ≤ 0.0175).

Figure 5 shows a slight tendency of increase in the bioreduction activity in cells after BSO treatment, as is more evident when cells were at lower density. The effective resazurin-reducing compound(s) in Ht22 is not clear, but the possible background from BSO was excluded. The mechanism behind the change is yet to be understood.

### Time course

**Figure 6:**
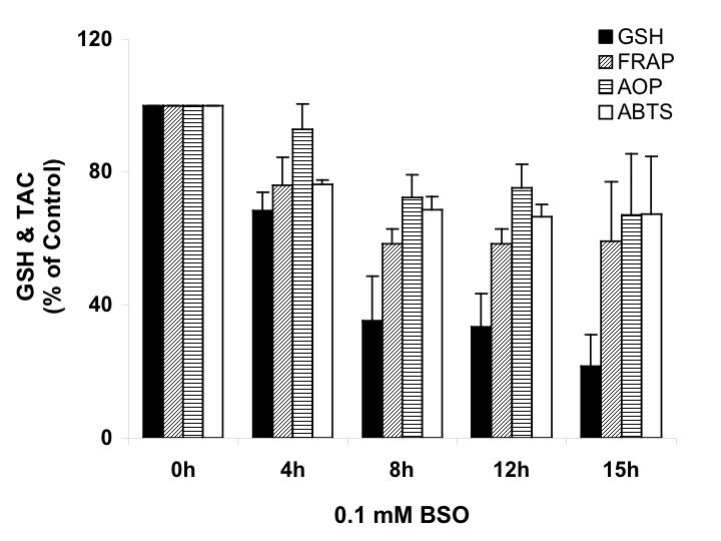
Time course of the responses of Ht22 cells to BSO treatment. The effects of total GSH level and total antioxidant capacities of Ht22 cells treated with 0.1 mM BSO for 4, 8, 12, and 15 hours were measured. Average value and SD are shown, N = 3. For total GSH, FRAP and ABTS, 4-hr treatment induced significant decreases *vs *control (P values ≤ 0.0001); from 4 hrs to 8 hrs, all of the assays showed significant decreases (P values ≤ 0.0326).

The responses of Ht22 cells to 0.1 mM BSO treatment were tested at 4, 8, 12 and 15 hrs, and results are expressed as percentage of 0 hr (Figure 6). For total GSH, FRAP and ABTS, 4-hr treatment induced significant decreases (P values ≤ 0.0001), while AOP value did not change significantly. From 4 hrs to 8 hrs, all of the assays showed significant decreases (P values ≤ 0.0326). Whereas from 8 through 15 hrs, no significant change was seen. The data suggests that for 0.1 mM BSO treatment, the majority of the GSH depletion occurred in the first 8 hrs, and that the depletion of other antioxidants were dynamically parallel to GSH depletion. Cell death was visible under microscopy after 20 hrs of 0.1 or 10 mM BSO treatments.

Seyfried et al. [12] found that PC12 cells treated with 0.5 mM BSO maintained 100 % of mitochondrial GSH in the first 4 hrs, while cytosolic GSH was completely depleted. Extending the treatment from 4 hrs to 6 hrs resulted in 50 % depletion of mitochondrial GSH. These data are in agreement with the significant decreases of total GSH level from 0 to 4 hrs, and from 4 hrs to 8 hrs in this study, although the differences in cell line and dosage of BSO in the two studies should be kept in mind while comparing the results.

### Antioxidant enzyme activities

#### Glutathione peroxidase

**Figure 7:**
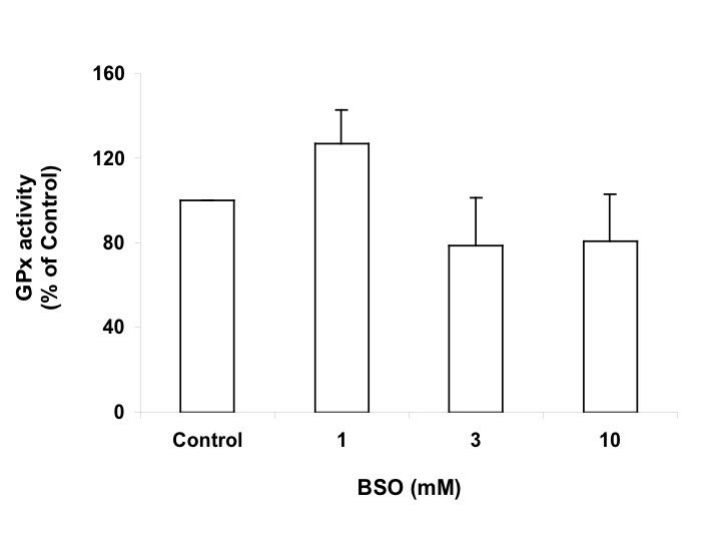
GPx activity of Ht22 cells treated with BSO for 15 hours. Average value and SD are shown, N = 3. Treatment with 1 mM BSO significantly increased the GPx activity *vs *control (P = 0.0401); and the decreases caused by 3 or 10 mM BSO are significant *vs *1 mM BSO treatment (P values ≤ 0.0421).

Glutathione peroxidases (GPxs) are selenium-containing antioxidant enzymes that reduce hydrogen peroxide to water, or lipid peroxides to ethanols, with GSH as reducing cofactor. Five isoforms of the GPx family are commonly known. In this study, the major activity detected by the assay is cellular GPx (GPx1). Figure 7 shows the change of GPx activity in response to 1, 3 and 10 mM BSO treatments for 15 hrs. Treatment with 1 mM BSO significantly increased the GPx activity to 127% of control (P = 0.0401). Treatment with 3 or 10 mM BSO decreased the activity by about 20% of control. This treatment was not significant compared to control, but is significant when compared to 1 mM BSO treatment (P values ≤ 0.0421). The results show that the regulation of GPx activity is dependent on the level of GSH depletion, and that the reserve pool of GSH may have critical function, since slight depletion caused significant changes.

#### Glutathione reductase

**Figure 8:**
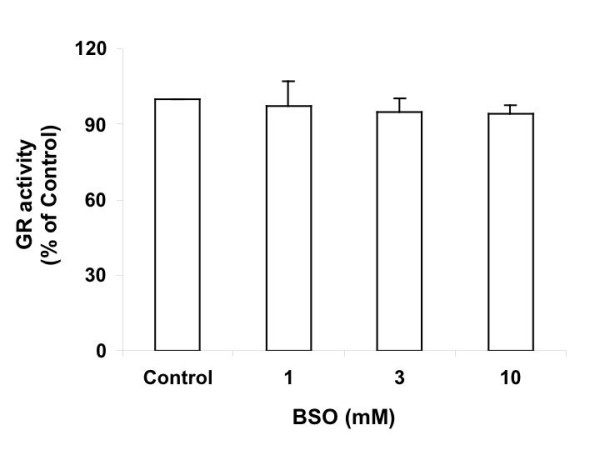
GR activity of Ht22 cells treated with BSO for 15 hours. Average value and SD are shown, N = 3. At 10 mM BSO concentration, the decrease is significant (P = 0.045) *vs *control.

Glutathione reductase (GR) reduces GSSG to GSH using NADPH as cofactor. When Ht22 cells were subjected to 1, 3 and 10 mM BSO treatment for 15 hrs, the GR activity in the cells dropped to 97%, 95% and 94% of control, respectively (Figure 8). At 10 mM BSO concentration, the decrease is significant (P = 0.045) in comparison to the control level. However, the toxicology cDNA expression array study in Section 5 did not show significant changes in the mRNA level of GR. The activity decrease of this enzyme may be due to enzyme inactivation caused by oxidative stress.

#### Superoxide dismutase

**Figure 9:**
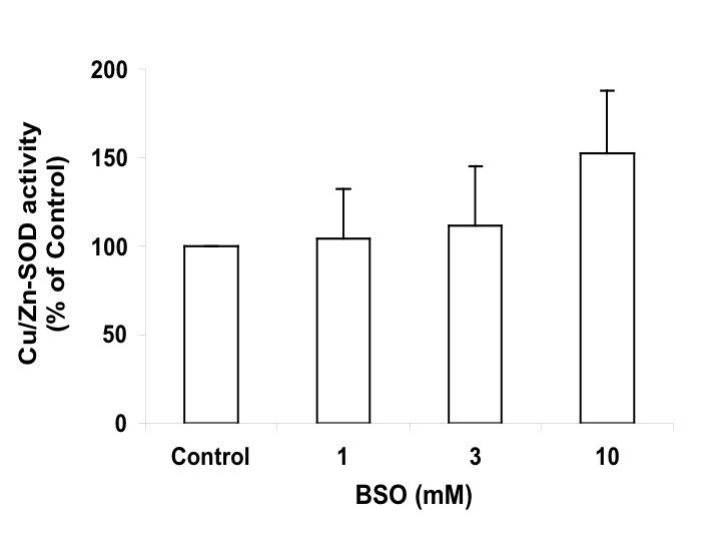
Cu/Zn-SOD activity of Ht22 cells treated with BSO for 15 hours. Average value and SD are shown, N = 3. Treatment of 10 mM BSO caused a trend of increase in SOD activity *vs *control (P = 0.0619).

Superoxide dismutase (SOD) converts superoxide anion to hydrogen peroxide, which can be further detoxified by GPx1 or catalase. Two isoforms of SOD are found in mammalian cells, known as Cu/Zn-SOD and Mn-SOD, and localized in cytosol and mitochondria, respectively. Incubating Ht22 cells with 1, 3, and 10 mM BSO for 15 hrs increased the Cu/Zn-SOD activity to 104%, 112%, and 152% (P= 0.0619) of the control level (Figure 9). The toxicology cDNA expression array study found a 2-fold increase of Cu/Zn-SOD mRNA level in response to treatment with 10 mM BSO treatment. In contrast to GPx, SOD responds more to severe GSH depletion or higher level of oxidative stress.

### Toxicology cDNA expression array

cDNA expression arrays are powerful tools for studying gene expression under various circumstances. In this study, we employed the Atlas rat toxicology array II (BD) to monitor the changes in gene expression after 15 hrs treatment with 10 mM BSO. Out of a total of 465 genes in the array, mRNA level of 3 genes were significantly decreased, and 10 significantly increased (Table 1). The increased mRNA level of heat shock protein (HSP, the induction of which correlates with the abundance of unfolded polypeptide chains) and eukaryotic peptide chain release factor 1 (ERF1, which functions in termination of translation) indicate the stress caused by BSO treatment affected proteins at translational and structural levels. The increased mRNA level of antioxidant enzymes Cu/Zn-SOD and thioredoxin peroxidase 2 (TPx II, a peroxidase that requires thioredoxin or thiol-containing intermediates to carry out its peroxidase function) suggests these enzymes have important functions in coping with the oxidative stress caused by GSH depletion. Furthermore, both of these enzymes have been shown to protect cells from different inducers of apoptosis [14,15], indicating that they may have contributed to maintaining high cell viability after BSO treatment in this study.

## Conclusions

Inhibition of glutamylcysteine synthetase in Ht22 cells by BSO revealed two pools of GSH in the cells, one susceptible to depletion by low concentration of BSO, and the other more resistant to depletion. TAC values measured by FRAP, AOP and ABTS methods showed parallel time courses to GSH depletion, but different dose-responses. The GSH depletion studied did not result in increases in GSH/GSSG ratio, lipid peroxidation, or cell death, but affected MTT-based cell viability. The antioxidant enzyme activities of GPx, GR and Cu/Zn-SOD were affected by the GSH depletion. The mRNA levels of HSP90-beta, ERF1, Cu/Zn-SOD and TPx II were significantly increased after 10 mM BSO-15 hr treatment.

## Methods

### Cell maintenance and treatment

Ht22 cells were fed with Dulbecco's Modified Eagle Medium (DMEM) with 10% fetal bovine serum (FBS) supplement, and cultured at 50–55% relative humidity (RH), in 5% CO_2 _at 37°C. For MTT-based cell viability assay and Resazurin-based bioreduction activity assay, cells were seeded at a density of 1 or 0.5 million cells per well (corresponding to 1.3e5 and 6.5e4 cells per square cm, respectively) in 48-well cell culture plates (Costar) 12 hrs before BSO treatment. For other assays, the cells were allowed to reach 80–90% of optical confluency (about 2e5 cells per square cm) in 100 mm cell culture dishes (Costar) before the BSO treatment.

### TAC, GSH/GSSG, MDA assays

In this study, three methods were employed for total antioxidant capacity assay. The AOP assay (Antioxidant Potential assay kit, Oxis) tests the ability of samples to reduce Cu2+ to Cu+ at physiological pH, (assayed according to manufacturer's instructions with minor adjustments). The FRAP assay [16] tests the ability of samples to reduce Fe3+ to Fe2+ at pH 3.6, a low pH that inactivates thiol antioxidants. The ABTS assay [17] tests the ability of samples to scavenge ABTS radical at physiological pH. Modified versions [18] of FRAP and ABTS assays were used in this study. GSH was assayed for total level as well as GSH/GSSG ratio using GSH/GSSG ratio assay kit (Oxis). Manufacturer's instructions were followed with minor adjustments. Lipid peroxidation in cells was assayed using malondialdehyde (MDA), a product of lipid peroxide decomposition, as an indicator. The assay was carried out using a Malondialdehyde assay kit (Oxis).

### Cell viabilities and bioreduction activity assays

A common method of testing cell viability is trypan blue staining. As a vital dye, trypan blue enters dead cells, distinguishing them from live ones. In this study, 0.4% (w/v) trypan blue-PBS solution was mixed with properly diluted cells at 5:1 ratio, and cell numbers were counted using a hemocytometer. Cell viability can also be reflected by dehydrogenase activity, which indicates the activity of mitochondria (Cell growth determination kit/MTT based, Sigma). Dehydrogenase converts MTT into purple MTT formazan, causing a colorimetric change that can be monitored photometrically. The bioreduction activity of cells was monitored by an in vitro toxicology assay kit (Sigma), based on a blue to red color change when the oxidoreduction dye, resazurin, is reduced by the bioreduction activity of the cells. Both MTT and Resazurin assays were carried out following the kit instructions.

### GPx, GR, and SOD activity assays

GPx activity assay was based on the classical principle [19] with optimization to the Ht22 cell lysis. The peroxide used in this study was t-butyl hydroperoxide (0.323 mM), the concentration of GSH was reduced to 1.875 mM, and the pH of the assay was increased to 7.6. The GR and SOD activities were assayed by corresponding kits from Oxis.

### Toxicology cDNA array assay

Atlas rat toxicology array II was purchased from BD, and the assay was carried out following the instruction manual.

### Statistical work

F-test in SAS procedure "Proc GLM" was used for statistical work.

## Authors' contributions

JC designed experiments, treated and harvested samples, optimized and participated in the antioxidant assays, participated in the toxicology cDNA array assay. ASH participated in the toxicology cDNA array assay. AY participated in harvesting samples, and the antioxidant assays. MJB contributed to conception and design, critical revision and final approval of the article.

## Figures and Tables

**Table 1:** Genes with significantly changed mRNA levels after BSO treatment. The results were detected by Atlas rat toxicology array II in Ht22 cells treated with 10 mM BSO for 15 hours.

**Protein/gene**		**Normalized Intensity of control**	**Normalized Intensity of BSO sample**	**Ratio**
***mRNA increased***				
heat shock cognate 71-kDa protein (HSC73; HSC70)	Y00054	1773	4780	2.70
eukaryotic peptide chain release factor subunit 1 (ERF1); TB3-1; C11 protein [rat homolog of human]	M75715 (human)	208	519	2.50
thioredoxin peroxidase 2 (TDPX2); thioredoxin-dependent peroxide reductase 2; heme-binding 23-kDa protein (HBP23)	D30035	1710	3989	2.33
78-kDa glucose-regulated protein precursor (GRP78); immunoglobulin heavy chain binding protein (BIP); steroidogenesis-activator polypeptide; HSPA5	M14050	66	154	2.33
60S ribosomal protein L6 (RPL6)	X87107	1932	4353	2.25
copper-zinc-containing superoxide dismutase 1 (Cu-Zn SOD1)	Y00404	1285	2813	2.19
proliferating cell nuclear antigen (PCNA); cyclin	Y00047	1463	3210	2.19
thymosin beta-10 (TMSB10; THYB10); PTMB10	M17698	3545	7637	2.15
high mobility group protein 2 (HMG2)	D84418	957	1976	2.06
heat shock 90-kDa protein beta (HSP90-beta); HSP84; HSPCB	S45392	7737	15881	2.05
				
***mRNA decreased***				
DNA topoisomerase IIB (TOP2B)	D14046	4146	1502	0.36
osteopontin	M14656	3630	1535	0.42
contrapsin-like protease inhibitor related protein; SPI-3 serine protease inhibitor	D00753	13996	5961	0.43
